# Development and Implementation Path of Kindergarten Stem Educational Activities Based on Data Mining

**DOI:** 10.1155/2022/2700674

**Published:** 2022-01-25

**Authors:** Liangfu Jiang

**Affiliations:** ^1^College of Educational Science of Hunan Normal University, Changsha 410081, China; ^2^Normal College of Hunan University of Arts and Sciences, Changde 41500, China

## Abstract

Early childhood education in China has given stem education constant attention and study. On the one hand, it has introduced many foreign research findings on stem education, such as curriculum practice, evaluation systems, teacher training, and so on; on the other hand, this paper investigates the localization implementation path of stem education based on the realities of kindergartens. This paper investigates the development and implementation path of kindergarten stem education activities using data mining, analyzes how the kindergarten stem education monitoring index system is developed and further improved using data mining algorithm, and determines the function path and mode of data mining algorithm in kindergarten stem education. It is expected to be used as a reference in the development and implementation of stem education and teaching activities. The development and implementation path of kindergarten stem educational activities based on data mining algorithm using data technology to realize continuous audit can not only improve the audit means and scope but also provide new research ideas for the expansion and innovation of audit work, which is useful in building a path model of kindergarten stem educational activities development and implementation.

## 1. Introduction

In recent years, China has seen a surge in stem education, which has piqued the interest and support of a growing number of people. Through the organic integration of science, technology, engineering, and mathematics, stem education is a project-based learning and problem-solving oriented curriculum organization that aims to cultivate the activity development innovation consciousness and innovation ability of kindergartens [1]. The application of the stem education concept in kindergarten teaching not only meets the needs of the kindergarten population and development, but it also meets the requirements of activity development of teaching reform. Early childhood education in China has paid close attention to and studied stem education. On the one hand, it has introduced many foreign research findings on stem education, such as curriculum practice, evaluation systems, teacher training, and so on; on the other hand, the localization implementation path of stem education has been explored based on the reality of kindergartens [2]. Children's education concept of stem focuses on cultivating students' comprehensive learning and practical ability. The application of tools in kindergarten classroom teaching can not only effectively improve the comprehensive quality and ability of kindergarten but also improve the teaching efficiency to a certain extent [3]. These explorations have accumulated rich resources, implementation paths, and experience for the current stem education in kindergartens [4, 5].

As a potential resource of STEM education in kindergartens, related research is still lacking. This paper takes this as a starting point to explore the development and implementation path of STEM education activities in kindergartens based on data mining [6]. Generally speaking, China's preschool children's science education has gone through the process of germination, stagnation, development, reference, localization, and scientific transformation, forming the curriculum system that emphasizes localization and local growth, the development goal of educational activities for the country, the curriculum content closely related to children's life, and the implementation path organization form that focuses on “doing”. At present, there are many theoretical researches on STEM educational concept in kindergartens in China, but the practical research on STEM educational activity development in kindergartens is not very rich [7]. Therefore, based on the data mining algorithm, this paper attempts to explore the science course “Make a paper plane” which is integrated with STEM concept, in order to provide more thoughts on the implementation path for front-line kindergarten teachers [8, 9]. The development and implementation path of STEM education activities in kindergartens is to comprehensively apply the techniques and methods of mathematical statistics, machine learning, and data mining to process and analyze STEM education data in kindergartens. Through data modeling, the correlation between learners' learning results and variables such as learning content, learning resources, and teaching behavior can be found to predict learners' future learning trends [10].

Under the background of the new era, education should pay attention to the cultivation of top-notch innovative strategic talents with international competitiveness, which is an effective way from “science and education” to “rejuvenating the country” and even “strengthening the country”. The development of stem educational activities in kindergartens, implementation of scientific educational activities in kindergartens' aims, and quality improvement [11]. Therefore, the research on the implementation path of the development of stem education activities in kindergartens is the objective demand to promote the development of stem education in preschool kindergartens. Ideal data mining algorithm for kindergarten stem education activity development and implementation path using data technology to realize continuous audit can not only enhance audit means and broaden audit scope but also provide new research ideas for the expansion and innovation of audit work, and help to build a kindergarten stem education activity development and implementation path model based on Data Mining [12, 13]. We can obtain valuable information in a variety of complex data using the data mining algorithm, which not only allows us to grasp problems that arise in the development of kindergarten stem education activities at any time, but also allows us to adjust current kindergarten stem education construction methods and objectives according to data technology [14, 15]. This paper analyzes how the kindergarten stem education monitoring index system is developed and further improved by using data mining algorithm, in order to obtain the role path and mode of data mining algorithm in kindergarten stem education. It is expected to be used as a reference in the development and implementation of kindergarten stem education activities.

## 2. Related Work

Bonneton-Botté et al. [16] suggest that teachers should adopt constructivism teaching strategies and refer to the precursor model of shadow formation, which has a positive effect on children's understanding and identification of physical phenomena of shadows. Through the method of big data analysis, in recent two years, more and more researchers have paid attention to STEM education in preschool stage, and most of them are foreign researchers, but the overall quantity is still small [17]. The research contents mainly focus on early childhood STEM education policies, kindergarten STEM courses, and kindergarten STEM teachers. Research shows that children can learn scientific concepts and vocabulary better by adopting reactive teaching or combining reactive teaching with explicit teaching [18]. Mcgoey et al. [19] point out that the practice of early science education is an effective strategy for science teaching and learning, and it is the foundation for the future development of kindergarten children's scientific literacy. Whiting et al. [20], through big data analysis [21], show that STEM education is very good at solving practical problems in real life. Therefore, teachers should observe their behaviors through daily contact with children, and then explore their interests and needs. Research shows that scaffolding instruction can help kindergarten and primary school teachers, as well as researchers, analyze the science education activities in kindergartens, and judge whether the adopted strategies are effective, which strategies need to be eliminated, and what other strategies may be needed [22]. Scannell et al. [23] point out that STEM education plays a significant role in improving national quality, stimulating employment and balanced income distribution, promoting ethnic equality and gender equality, strengthening national economic strength and driving innovation, etc., and it is a booster of American national competitiveness. Through the method of big data analysis, in children's daily life, although some contents are not initiated by young children, they are initiated by adults, but they play a great role in children's growth and habit formation, which requires teachers to pay more attention [24]. Research shows that experimental basic education has a positive impact on the ability of 5-year-old children to solve scientific problems [25]. Experiment-based science education projects include experiments aimed at improving children's scientific process skills, independent thinking, decision-making, and problem-solving process. Literature [1] pointed out that the poor status of STEM education at the present stage is mainly influenced by the high cost of university education, poor academic preparation, demographic factors, lack of local core staff, and so on.

This paper studies the development and implementation path of kindergarten stem education activities based on data mining, and finds that kindergarten stem activities and project-based learning fit well, and project-based learning provides a good platform and opportunity for stem education.

## 3. Principle and Model of Data Mining Algorithm

**Figure 1:**
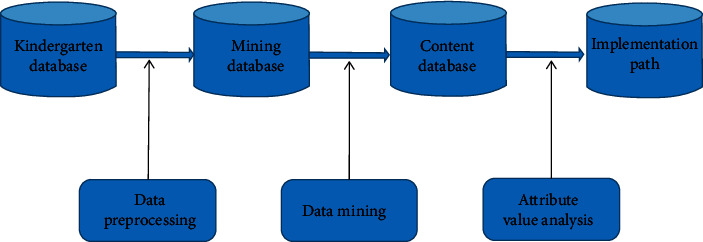
Data mining model.

Data Mining is a new discipline of “developing methods and exploring unique types of data in the educational environment, so as to better understand students and their learning environment through these methods.” Data mining (DM) is a process of discovering interesting knowledge from a large amount of data stored in databases, data warehouses, or other information bases, and it is a marginal discipline involving database management, artificial intelligence, machine learning, pattern recognition, data visualization, and other disciplines. If we can dig out this hidden pattern from the development of STEM education activities in kindergartens, its significance is self-evident. We can describe the general process of kindergarten data implementation path as shown in Figure 1.

### 3.1. Establish Data Mining Database

Analyze the data characteristics of the existing kindergarten information system, and establish the mining database.

### 3.2. Data Preprocessing

For the activity development and implementation path-related data of the existing stem education information in the kindergarten, the sequence mining method is used for standardized processing to ensure the integrity and consistency of the data, smooth the noise data, identify and delete outliers, so as to improve the data quality, improve the mining accuracy and performance, and store the processed data in the mining database.

### 3.3. Design Mining Algorithm

A general mining algorithm for a single disease can be designed by combining clustering analysis and ant colony algorithm in bionic algorithm. Clusters of related diseases can be obtained by clustering, and ant colony algorithm can be used to optimize the formation of clusters, so as to form an optimized single path.

### 3.4. Establish the Implementation Path Database

According to the related concepts of stem education in kindergartens, the adult attributes and their constraints are explained, and the content database storing the extracted values is established. Through the corresponding expression, it will be transformed into the familiar implementation path.

**Figure 2:**
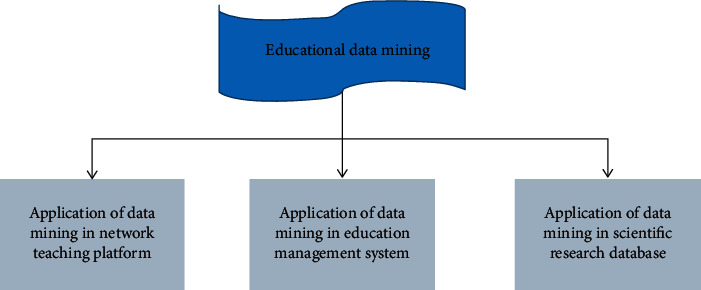
Distribution of educational data mining applications.

To construct the development and implementation path of STEM education activities in kindergartens from the perspective of data mining, it is necessary to realize it with the help of information active push technology. The basic activities of education are often divided into three categories: teaching, management and scientific research, because there are some differences in business processes and objects of concern in various fields. Therefore, the application of data mining in education should also be divided into three areas, as shown in Figure 2.

**Figure 3:**
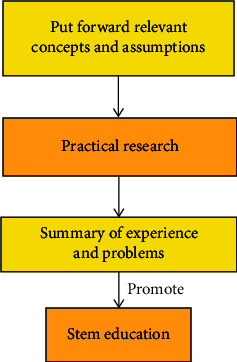
STEM educational research model.

In the application of information active push technology, it is usually through SMS notification and server client connection, which is not only conducive to better let the majority of teachers and students know the school related teaching information and management information, but also make use of personalized data mining technology and information customization technology to continuously improve the efficiency of information personalized service. The stem education research model is summarized as the “win-win” model of stem education research, as shown in Figure 3.

STEM education research model is not only conducive to obtaining more extensive and effective data, but also indirectly promotes STEM education concept and its culture. At the same time, this method is also helpful to strengthen inter-regional cooperation, improve the imbalance of inter-regional education development, and improve the quality of education in underdeveloped areas.

**Figure 4:**
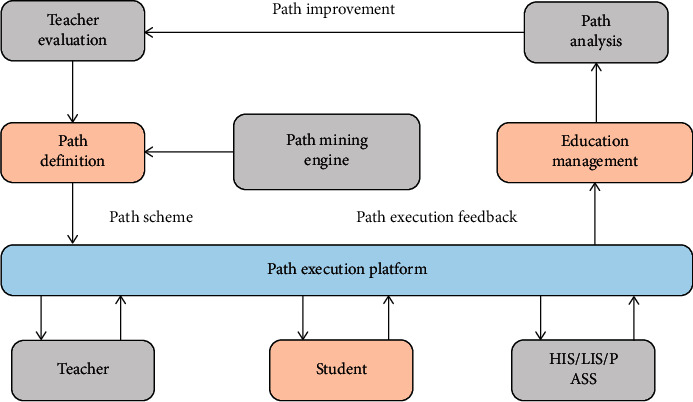
Implementation of route information system operation scheme.

Path informatization is realized on the basis of the development of existing business system in kindergarten STEM education activities. The implementation of path system does not change the operation of existing system in kindergarten, but seamlessly links with it to realize the interaction and sharing of information. The operation scheme of the implementation path system is shown in Figure 4.

In this paper, *K*-means, a data mining algorithm based on partition, is used for text data mining. The advantage of *K*-means is simple and fast. The basic principle of *K*-means algorithm is as follows: assuming that the extracted original data set is *X*_*i*_ = (*X*_1_, *X*_2_,..., *X*_*N* − 1_, *X*_*n*_), the purpose of *K*-means is to segment the original data set *X* into *k* categories under the condition of given clustering number *k* (*K* ≤ *n*). The *k*-means algorithm is described below.

Assume that the data set to be classified is *X*_*i*_ = {*X*_1_, *X*_2_, ... *X*_*N* − 1_, *X*_*n*_}, the output clusters are *C*_*j*_ = {*C*_1_, *C*_2_, ..., *C*_*K*_} (1 ≤ *J* ≤ *K*, *K* ≤ *N*), and the center value of cluster CJ is *M*_*J*_ = {*M*_1_, *M*_2_, ... *M*_*K*_}.

(1) $$\begin{array}{c} Mj=\frac{1}{n_{j}}\sum_{X_{i}\in C_{j}}X_{i}, \end{array}$$

where *N*_*J*_ represents the number of input data *X*_*i*_ contained in the output cluster *C*_*J*_. Euclidean distance is used to calculate the distance between a word vector and the centroid. Since *X*_*i*_ and *M*_*J*_ are *n*-dimensional vectors, it is necessary to use Σ to calculate the Euclidean distance between two *n*-dimensional vectors

(2) $$\begin{array}{c} \text{distance}=\sqrt{\sum_{i,j=1}^{n}{\left\|X_{i}-M_{j}\right\|}^{2}.} \end{array}$$

It is mainly used to extract keywords and abstracts for text, and its core ideas are as follows:

(3) $$\begin{array}{c} \text{WS}\left(V_{i}\right)=\left(1-d\right)+d*\sum_{V_{j}\in In\left(V_{i}\right)}\frac{w_{ji}}{\sum_{V_{k}\in \text{Out}\left(V_{j}\right)}w_{jk}}\text{WS}\left(V_{j}\right). \end{array}$$

Assuming that data mining has been completed, there are *n* clusters, in which there are *m* points in cluster I, then the calculation process of Silhouette index is as follows

**Step 1 .**

calculate the intracluster distance *a*(*i*, *j*) for the *J*-th point in cluster *I*, where *a* (*i*, *j*) is the average distance between any data object *J* in cluster *I* and other data objects in the cluster

(4) $$\begin{array}{c} a\left(i,j\right)=\frac{1}{M-1}\sum_{k=1,k\neq j}^{M}\text{Distance}\left(j,k\right). \end{array}$$

**Step 2 .**

for the *j*-th point in cluster *I*, calculate the cluster spacing *b* (*i*, *j*). *b* (*i*, *j*) is the minimum of the average distance from any data object *J* in cluster *I* to any other data object in cluster *I*

(5) $$\begin{array}{c} b\left(i,j\right)=\underset{k=1⟶N,k\neq i}{\text{min}}\left(\text{Avg Distance}\left(j,k\right)\right). \end{array}$$

**Step 3 .**

calculate the Silhouette index of point *J* in cluster *I*.

(6) $$\begin{array}{c} \text{Silhouette}\left(i,j\right)=\frac{b\left(i,j\right)-a\left(i,j\right)}{\text{max}\left\{a\left(i,j\right),b\left(i,j\right)\right\}}. \end{array}$$

**Step 4 .**

calculate the Silhouette index of cluster *I*.

(7) $$\begin{array}{c} a\text{vg\_Silhouette}\left(i\right)=\frac{1}{M}\sum_{j=1}^{m}\left(\text{Silhouette}\left(i,j\right)\right). \end{array}$$

**Step 5 .**

calculate the Silhouette index of the whole data set.

(8) $$\begin{array}{c} \text{avg\_SILHOUETTE}=\frac{1}{N}\sum_{i-1}^{N}\left(a\text{vg\_Silhouette}\left(i\right)\right). \end{array}$$

The implementation path of the specified disease is excavated by the implementation path mining engine in this scheme, which is then put into teaching application after teacher evaluation and revision, resulting in a specific teaching scheme and teaching record. Then, to record the path's implementation, interact with the kindergarten systems. Finally, the feedback data is sent to the department in charge of handling abnormal alarms, abnormal monitoring, and statistics, as well as preparing for path analysis and optimization. The Data Mining Society defines educational data mining as “a new discipline that analyzes unique and growing large-scale data in the educational environment through development methods,” which is similar to Baker's definition. Data mining is not only useful for improving the teaching and learning experiences of teachers and students, but it also has the potential to support scientific management decisions and strengthen school governance. In the data age, data mining meets the practical needs of mining the value of educational data and discovering the educational data implication law.

## 4. Development and Implementation Path of Stem Educational Activities in Kindergartens

### 4.1. Development and Implementation Path of Kindergarten Stem Education Activities Based on Data Mining

To carry out STEM education projects in kindergartens, first of all, teachers are required to have STEM concept and consciousness, and be able to guide their own specific practice by this, and to use STEM vision to discover the value contained in the projects, so as to effectively guide the activities. STEM education in kindergartens and educational research and practice activities are destined to be full of value, complexity, creativity, and uncertainty. In the research and practice of kindergarten STEM education, although data science or data mining can tell people what some phenomena and problems in kindergarten STEM education are, the present situation and state of kindergarten STEM education are, it cannot explain such valuable problems as “why is this so” and cannot put forward countermeasures and suggestions as “how to do” according to the characteristics and development rules of kindergarten STEM education activity development and kindergarten STEM education implementation path practice. The development and implementation path of STEM education activities in kindergartens has the characteristics of cross-domain, involving the study of science, technology, mathematics, and other fields of knowledge. Second, both of them pay attention to children's life experience. Based on the characteristics of children's thinking in concrete images, they emphasize the simplicity and interest of activities, and focus on exploration and operation, so that children can gain direct experience. Third, both of them often involve real problems in activities. Children need to communicate and discuss with peers and teachers in activities to form a small learning community, from which they can help each other to get useful experience and guidance, and then learn to solve real problems step by step.

Data mining is a novel situation and backdrop for the development and implementation of stem education activities in kindergartens, but it comes with its own set of challenges and shortcomings, including low data density, research ethics, personal privacy, and data security. While appreciating the value of data mining, we must also recognize its drawbacks and limitations. If children want to create a mental “primary school,” they must first determine the construction project, then design and plan the school project in the form of painting, and finally construct the school. During the construction process, children must consider which materials to use, what to build first, what to build again, which method to use, how to solve balance and stability problems, and so on. All of these issues are related to engineering and technical education.

A series of practical researches on STEM project activities based on data mining algorithm were carried out. Under the guidance of the special research project “Research on STEM Education Practice in Kindergarten Based on Project Learning” of Shaanxi Academic Leaders Training Plan, the exploration with project learning as the carrier was completed.

1. Established the basis for the content selection of STEM education activities in kindergartens. Being close to children's life is a real problem that children are interested in and permeates all aspects of children's daily life.
2. Summarize the implementation path of STEM education activities in kindergartens.
3. Complete the compilation of Case Collection of STEM Education Activities, which contains 15 activity cases. The exploration at this stage helps us sort out the selection basis and implementation path of STEM education activities, and lays a solid foundation for the follow-up kindergarten STEM curriculum construction.

When screening children's interesting questions, teachers should be able to quickly judge whether the problem comes from children's life or impulse; judge whether the problem has the essence of science, mathematics, and technology; and judge whether the combination of children's teams can be used to solve problems. Stem education is a comprehensive science education based on constructivism, “learning by doing” and other theories. Developing stem education in early childhood has become a field of concern for researchers and early childhood educators at home and abroad. Integrating stem education concept into kindergarten education has become a new exploration direction. Generally speaking, data mining information collection is an organic combination of Official Statistical Organization database, daily management database of policy implementation department and field survey database. Therefore, through the data mining application path and method of developing and implementing the path monitoring index system in kindergarten stem education activities, China should strengthen the construction and analysis of data mining information in education monitoring, supervision, and evaluation; step up the construction of national education big data information center or database; and classify all kinds of Education data and information at all levels in China, improve the collection, analysis, and processing of relevant data of education monitoring index system at all levels, and build a data mining collection mechanism for kindergarten stem education activities in China. Real situations are often typical problem situations. After children experience complex and real problem situations, they will explore and understand knowledge and learn to transfer the understood knowledge to life. The development of stem educational activities in kindergartens often involves the real problems encountered by children. Children continue to push forward the process of activities through continuous problem exploration, solution, and implementation path.

The goal of the kindergarten STEM education activity based on data mining algorithm is to teach children how to effectively combine various disciplines to improve their problem-solving abilities. These various subject knowledge requirements are the foundation of STEM education activities in kindergartens and are required for solving problems. However, mathematics and science have distinct characteristics, and they can only be effectively integrated through engineering and design. As a result, we must focus on engineering and implementation path as the premise for STEM education activities in kindergartens. Garden A, with the support of the education administrative department and Shanghai Steiner Science Education Research Center, is implementing STEM project-based learning in kindergartens and, later, maker activities to broaden children's scientific and technological vision and cultivate their logical thinking, as part of the “Three-year Plan of Action for Preschool Education” and the second kindergarten curriculum reform. The development of kindergarten STEM education activities is a problem-solving activity, driven by the problems that children are most concerned about at the time, with a focus on the exploration and solution of practical problems in kindergarten STEM education, with an emphasis on children's understanding of knowledge and practical application on this basis.

### 4.2. Results and Analysis

**Figure 5:**
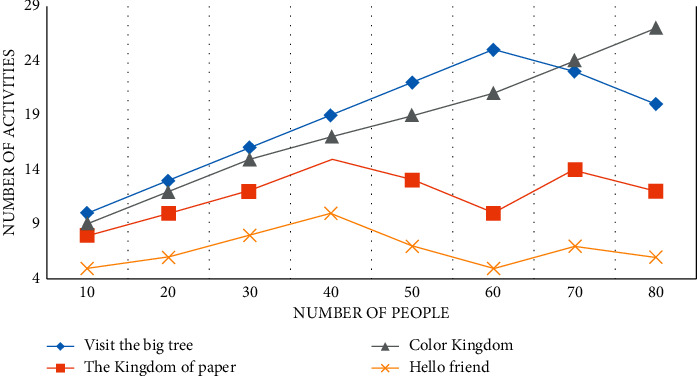
A number of theme activities in kindergarten during the first month.

**Figure 6:**
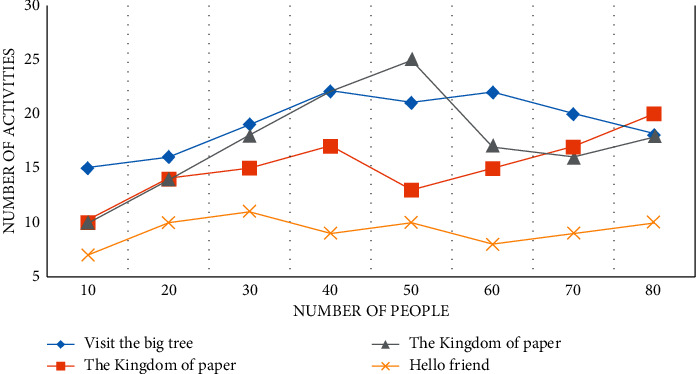
A number of theme activities in kindergarten for the second month.

**Figure 7:**
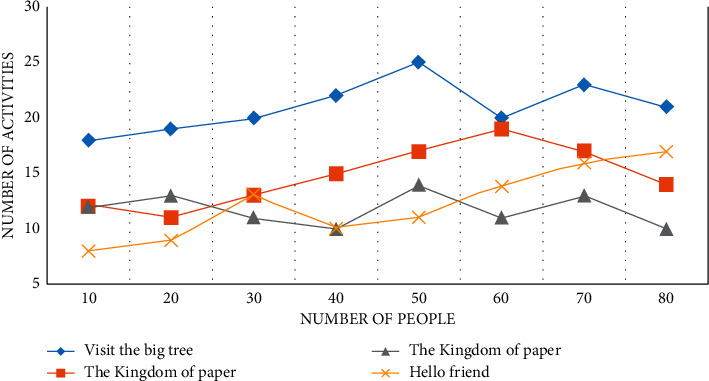
A number of theme activities in kindergarten for the third month.

Through the three-month investigation in kindergarten A, it is found that kindergarten A carries out activities in the form of themes, teaching around four theme activities in a semester, each theme lasts for two months, and each theme has about 50 collective activities, as shown in Figures 5–7.

The theme activity is “Visiting the Big Tree,” as shown in Figures 5–7, with 11 collective activities accounting for about 24.5% of the total. The Kingdom of Paper has nine collective activities, which account for about 16% of all activities. Because the focus of teaching at the beginning of kindergarten is to guide children in adapting to their new environment and cultivating their basic life skills, the theme “Hello Friends” consists of ten collective activities, resulting in a lack of scientific education activities in the first theme. Color Kingdom has 20 collective activities, accounting for 18.5% of the total. The quantitative analysis of science education activities in kindergarten A reveals that the design of science education activities in kindergarten A is quantitatively balanced with the design of activities in other fields, taking into account the setting of courses in five fields, which is more beneficial to children's overall physical and mental development and also meets the Guide and Outline requirements. The key factor that determines the smooth progress of science education activities, as well as the effect of the entire activities and the direction of children's development, is whether the goal of science education activities is reasonable or not.

**Figure 8:**
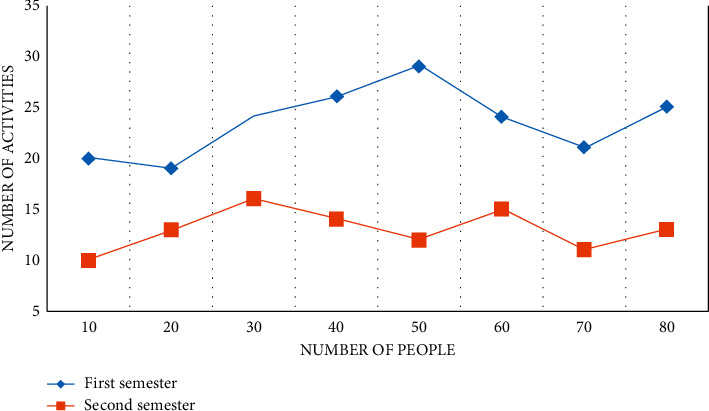
Distribution of “overall observation situation” frequency of small class teachers.

**Figure 9:**
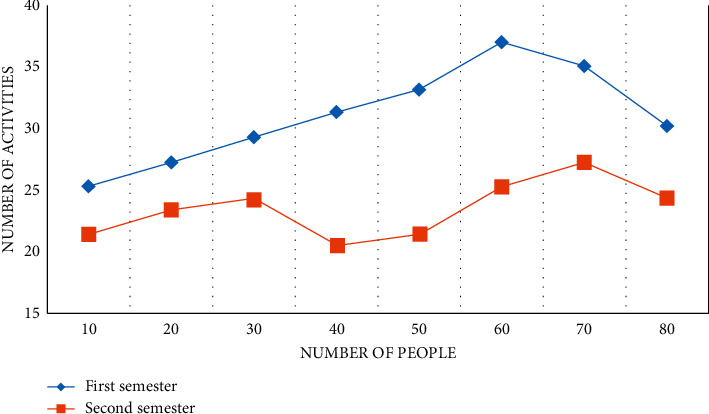
Distribution of “overall observation situation” frequency of middle-class teachers.

**Figure 10:**
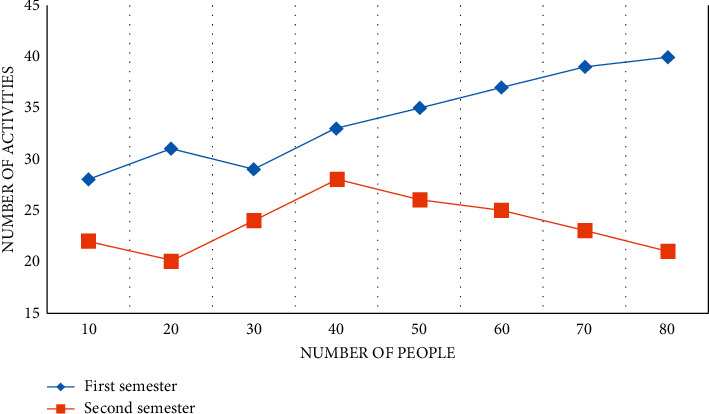
Distribution of “overall observation situation” frequency of large class teachers.

In this study, “overall observation situation” mainly refers to the main activity links of one-day life in kindergartens. The hypothesis of the study is: through action research, teachers' overall observation situations are becoming more and more diverse, and can infiltrate mathematics education and observation into all links of children's daily life. The following data shows the distribution of “overall observation situation” frequency of small class, middle class, and large class teachers, as shown in Figures 8–10.

From the above data, it can be found that according to the chi-square test conditions, when comparing the composition ratio of two samples, this study needs to process the data and combine the frequencies in some columns to meet the test requirements. From the above data, we can see the main situations of observation in the first semester. The hypothesis of this study is that after action learning, teachers can naturally integrate the observation of children's mathematics learning into various situations of daily life. In this study, observation situations are collectively referred to as “other situations,” and the data in these situations are combined to test whether the overall observation situation distribution of small class teachers has changed significantly.

According to the aforementioned data, the main situation observed by middle-class teachers in the first semester is “game activity.” The hypothesis of this study is that by incorporating observation of children's mathematics learning into various situations in children's day-to-day lives, preschool teachers can naturally integrate observation of children's mathematics learning into various situations in children's day-to-day lives. The observation situation is divided into “other situations” in this study, and the data from these situations are combined to see if the distribution of middle-class teachers' observation situations has changed significantly. In other words, the composition ratio of middle school teachers' “overall observation situation” did not change significantly from the first semester to the second semester. It is clear that, whether in the first or second semester, middle-class teachers' observations are primarily focused on “game activities.”

From the above data, it can be found that from the first semester to the second semester, the “observation content” of large class teachers has changed significantly. Preschool teachers tend to “observe the learning results” in the first semester, which is related to observation habits developed over time; on the other hand, it is possible that the connotation of “evaluation of early childhood development” is not well understood, and there is no link between “evaluation” and “more effective early childhood learning support.” During the second semester, most teachers are able to observe the children's mathematics learning process thoroughly and meticulously and record the important information learned. The details not only describe “what children learn” but also “how children learn,” according to the records. The research and implementation path for kindergarten STEM education is distinct. Data alone will not be able to solve all of the problems in kindergarten STEM education research and implementation. As a result, we should promote the reform and development of STEM education activities development and implementation path in kindergartens by using data mining as a new way of thinking and path, research paradigm and method, and practical tools and means based on STEM pedagogy.

## 5. Conclusions

To summarize, the article believes that correct ideas and reasonable methods are required for the actual implementation of stem education in kindergartens. Preschool teachers should also work on improving their professional quality in stem education. In the era of data mining, objectively analyzing the various paths and methods of data mining in the field of education is beneficial to our clearer and objective cognition of data mining, as well as thinking and using big data based on educational research and practice. As a result, China should strengthen the construction and analysis of data mining information in educational monitoring, supervision, and evaluation, as well as accelerate the construction of a national education big data information center or database, by developing stem educational activities in kindergartens and implementing data mining application paths and methods of path monitoring index system. The role and impact of data mining on education are discussed in this paper, which is based on data collection, acquisition, processing, and analysis. The stem education monitoring index system constructs the data hierarchy and category model of the index, analyzes the data collection mechanism of the index, and collects and obtains the data of each index of the index system, based on large-scale data investigation and database, using relevant ideas and methods of data mining. The development of stem educational activities in kindergartens frequently involves real-life issues that children face. Through a continuous problem exploration, solution, and implementation path, children continue to push the activity process forward.

## Data Availability

The data used to support the findings of this study are included within the article.
